# Supplementary material for: Integrative multiplatform molecular profiling of benign prostatic hyperplasia identifies distinct subtypes
Source: Nat Commun. 2020 Apr 24;11:1987. doi: 10.1038/s41467-020-15913-6 (PMC7181734; doi:10.1038/s41467-020-15913-6)
Supplement: Supplementary file 1 — Supplement Information [file 41467_2020_15913_MOESM1_ESM.pdf]

Integrative multiplatform molecular profiling of benign prostatic hyperplasia identifies distinct subtypes

Deli Liu, et al.

Supplementary figures

Supplementary Figure 1. Histological images of BPH cases from subgroups A and B.
Supplementary Figure 2. Circos plots of 18 BPH samples.
Supplementary Figure 3. *ERG* gene expression in BPH, normal control samples, prostate cancer samples with *ERG* fusion, and samples without *ERG* fusion from TCGA study[1].
Supplementary Figure 4. IGV snapshot of *TMPRSS2-ERG* fusion in prostate cancer samples with *TMPRSS2-ERG* fusion from TCGA study[1], BPH and normal control samples.
Supplementary Figure 5. The hierarchical clustering and heatmap of BPH subgroups based on BPH transcription signature.
Supplementary Figure 6. BPH transcription signature is not transitional zone specific.
Supplementary Figure 7. Heatmap of BPH and control samples based on prostate AR target genes[2].
Supplementary Figure 8. Differentially methylated CpGs and regions found in BPH samples by ERRBS data.
Supplementary Figure 9. Hierarchical clustering and heatmap of 18 BPH samples based on prostate stromal signature[3].
Supplementary Figure 10. Hierarchical clustering and heatmap of GSE101486 samples based on prostate stromal signature[3].
Supplementary Figure 11. Beeswarm plot of stromal enrichment difference between BPH subgroups.
Supplementary Figure 12. Spider chart of normalized enrichment score of stromal signatures from single-cell RNA-seq[4] and bulk RNA-seq data[5] in BPH subgroups.
Supplementary Figure 13. Metabolism dysregulation between two subgroups from both current and GSE101486 studies via GSEA.
Supplementary Figure 14. CONSORT diagram detailing workflow for clinical study.
Supplementary Figure 15. Boxplot of prostate size changes on patients with and without *mTOR* inhibitors.

Supplementary Tables

Supplementary Table 1: Clinical annotations of BPH samples.
Supplementary Table 2: Mutation results of BPH samples.
Supplementary Table 3: Small indel results of BPH samples.
Supplementary Table 4: Recurrent amplifications of BPH samples.
Supplementary Table 5: Recurrent deletions of BPH samples.
Supplementary Table 6: No known mutations from primary prostate cancer found in BPH WGS/WES data.
Supplementary Table 7: Clinical information of RNA-seq control samples.
Supplementary Table 8: No known mutations from primary prostate cancer found in BPH RNA-seq data.
Supplementary Table 9: Transcriptional signature between BPH and control samples.
Supplementary Table 10: Promoter methylation signature between BPH and control samples.
Supplementary Table 11: Transcriptional signature between two BPH subgroups.
Supplementary Table 12: GSEA output of metabolism related signatures from subgroup BPH-A when compared to the other subgroup from current study, and from one subgroup when compared to the other subgroups from GSE101486 study.
Supplementary Table 13: Transcriptional signature between BPH-A subgroup and control samples.
Supplementary Table 14: Transcriptional signature between BPH-B subgroup and control samples.
Supplementary Table 15: GSEA output difference of hallmark signatures from two BPH subgroups when compared to control samples.
Supplementary Table 16: Nominated compounds from each BPH subgroup when comparing with control samples across multiple cell lines, and summary from all cell lines via Connectivity Map (CMAP).

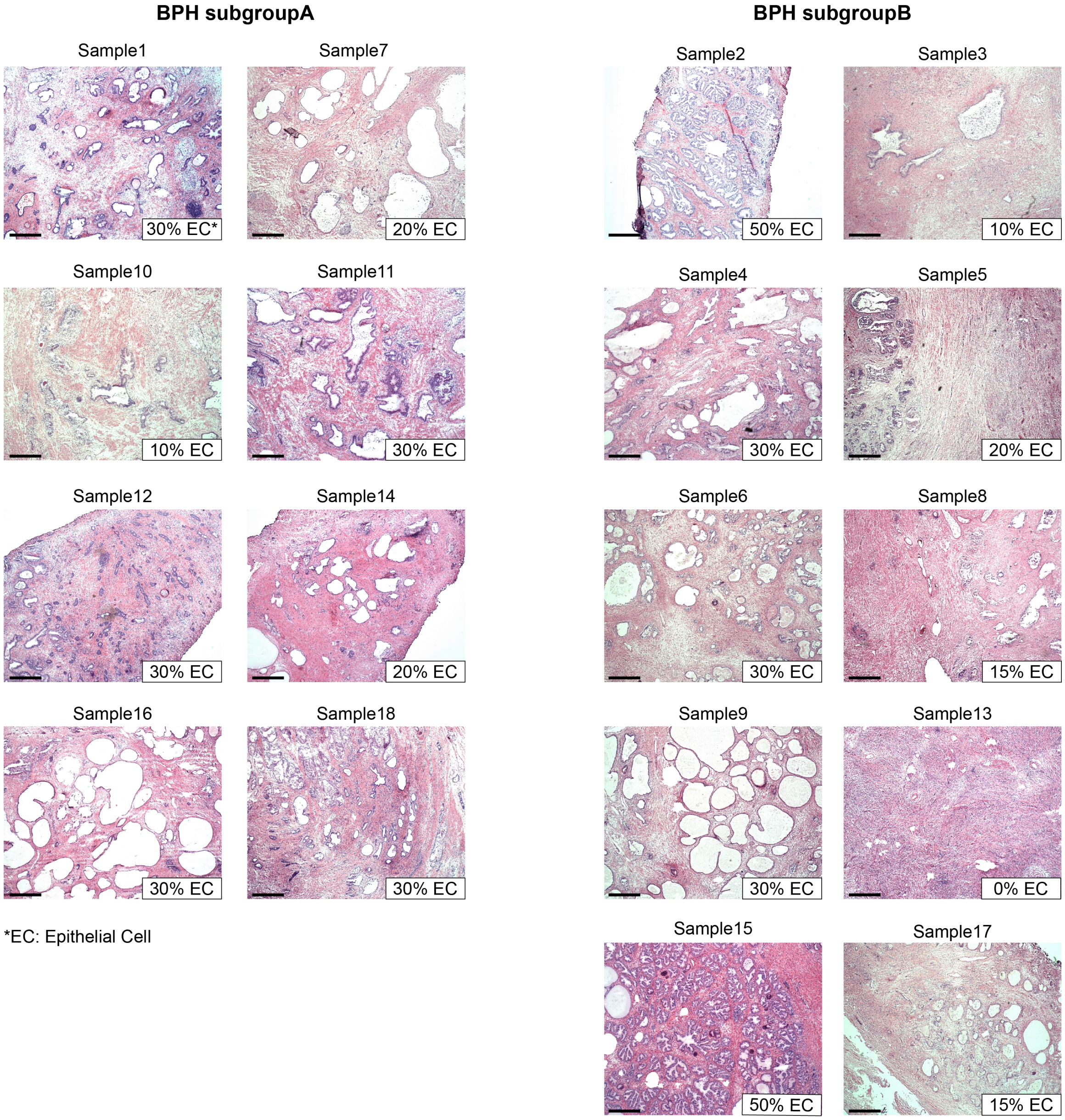

Supplementary Figure 1. Histological images of BPH cases from subgroups A and B, with percentage of epithelial cells shown at the bottom right. 100x original magnification, scale bar 100 μm.

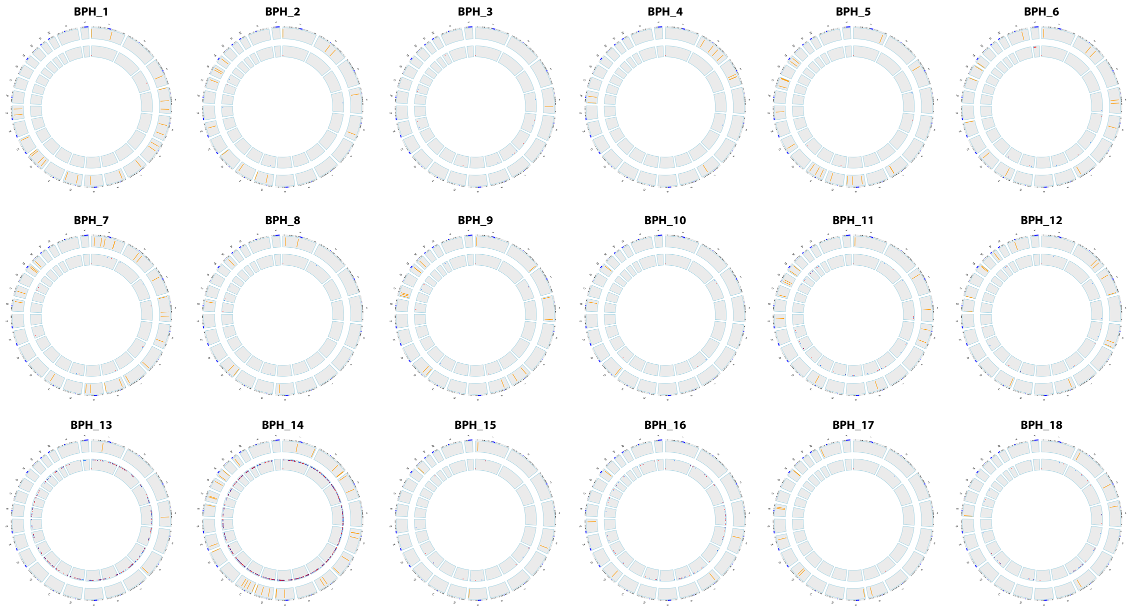

Supplementary Figure 2. Circos plots of 18 BPH samples. The rings from outer to inner represent somatic coding mutations, copy number alterations and fusion genes respectively.

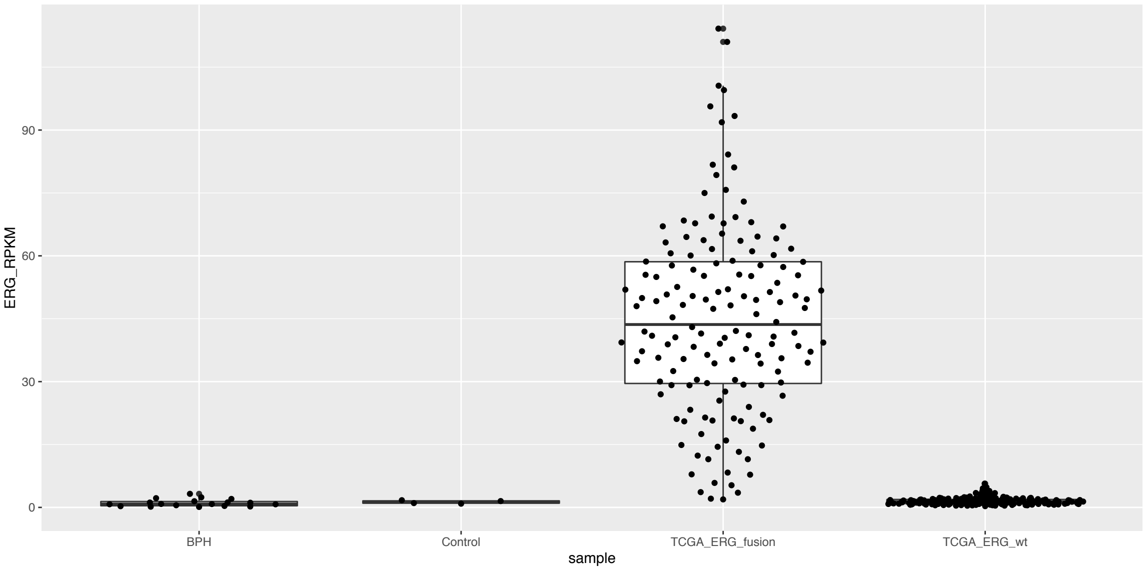

Supplementary Figure 3. *ERG* gene expression in BPH (n=18), normal control samples (n=4), prostate cancer samples with *ERG* fusion (n=152), and samples without *ERG* fusion (n=181) from TCGA study[1]. Definition of box plots: the center line represents median value, box limits represent 25% and 75% quantiles, and the top and bottom lines represent minimal and maximal values, respectively.

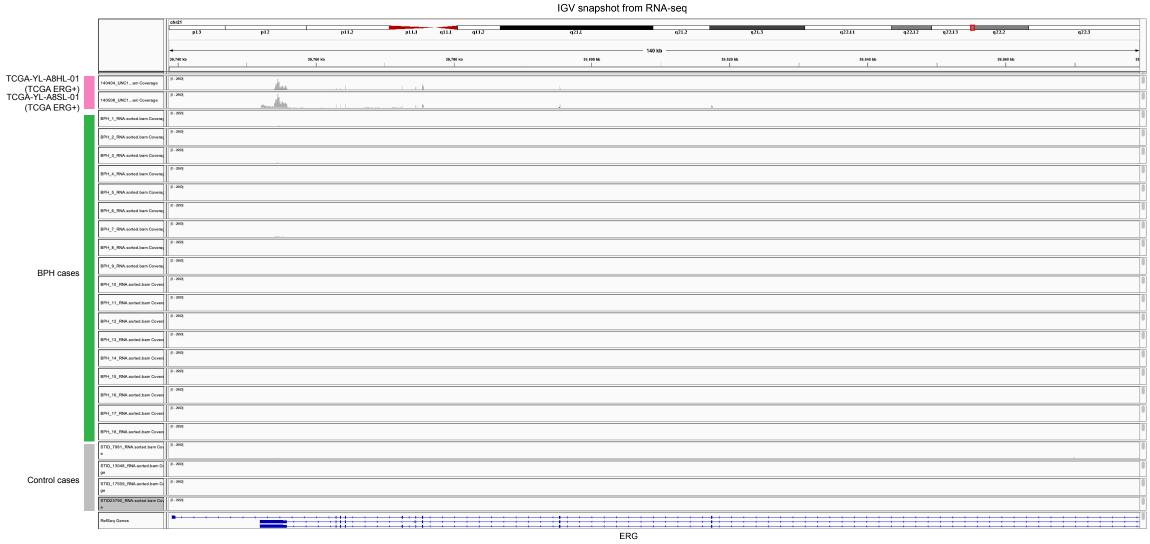

Supplementary Figure 4. IGV snapshot of *TMPRSS2-ERG* fusion in prostate cancer samples with *TMPRSS2-ERG* fusion from TCGA study[1], BPH and normal control samples.

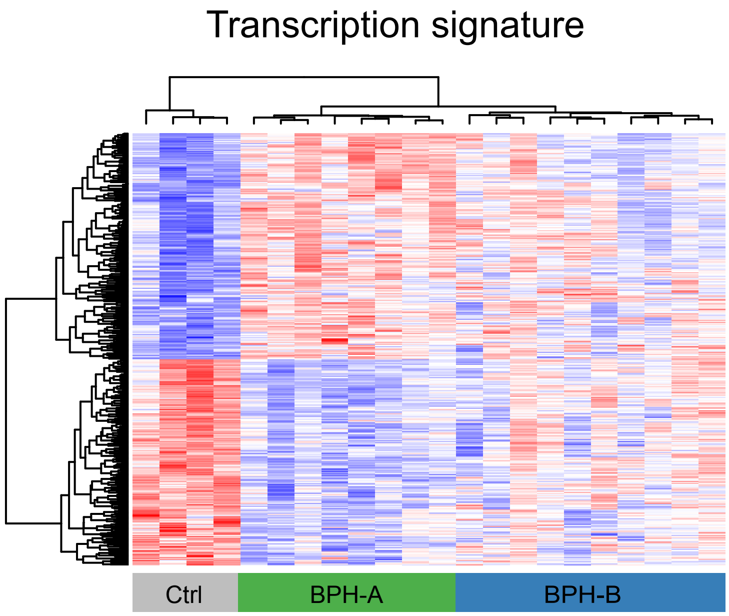

Supplementary Figure 5. The hierarchical clustering and heatmap of BPH subgroups based on BPH transcription signature from Figure 2B.

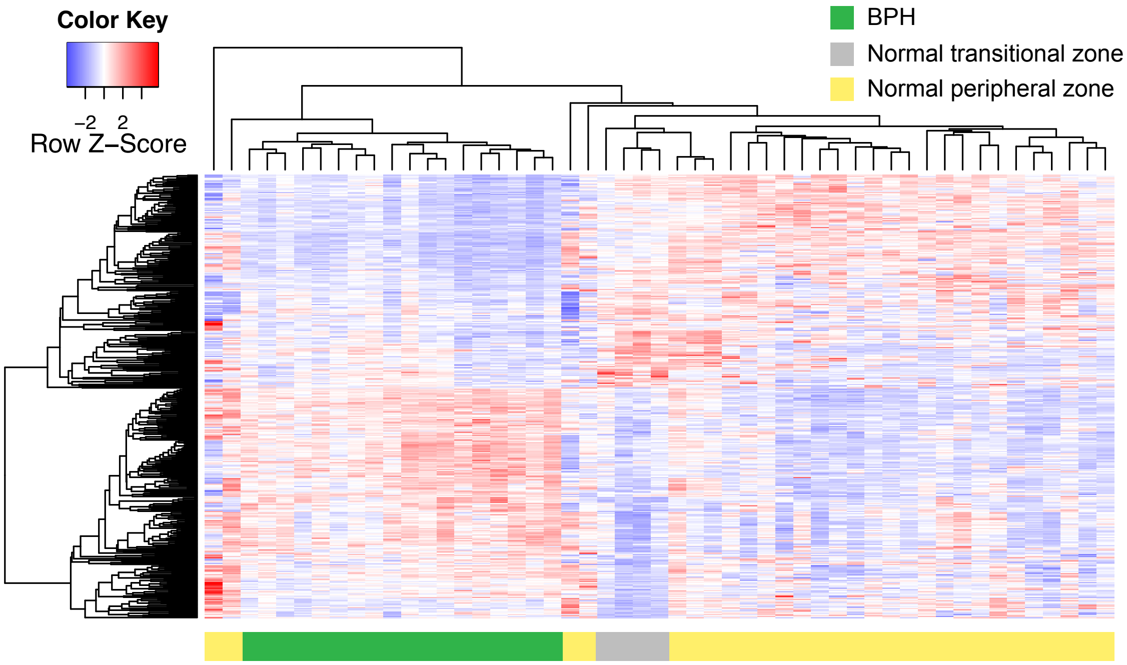

Supplementary Figure 6. BPH transcription signature is not transitional zone specific. The hierarchical clustering and heatmap of 18 BPH, 4 normal transitional zone, and 29 normal peripheral zone samples[6, 7, 8], based on BPH transcription signature from Figure 2B.

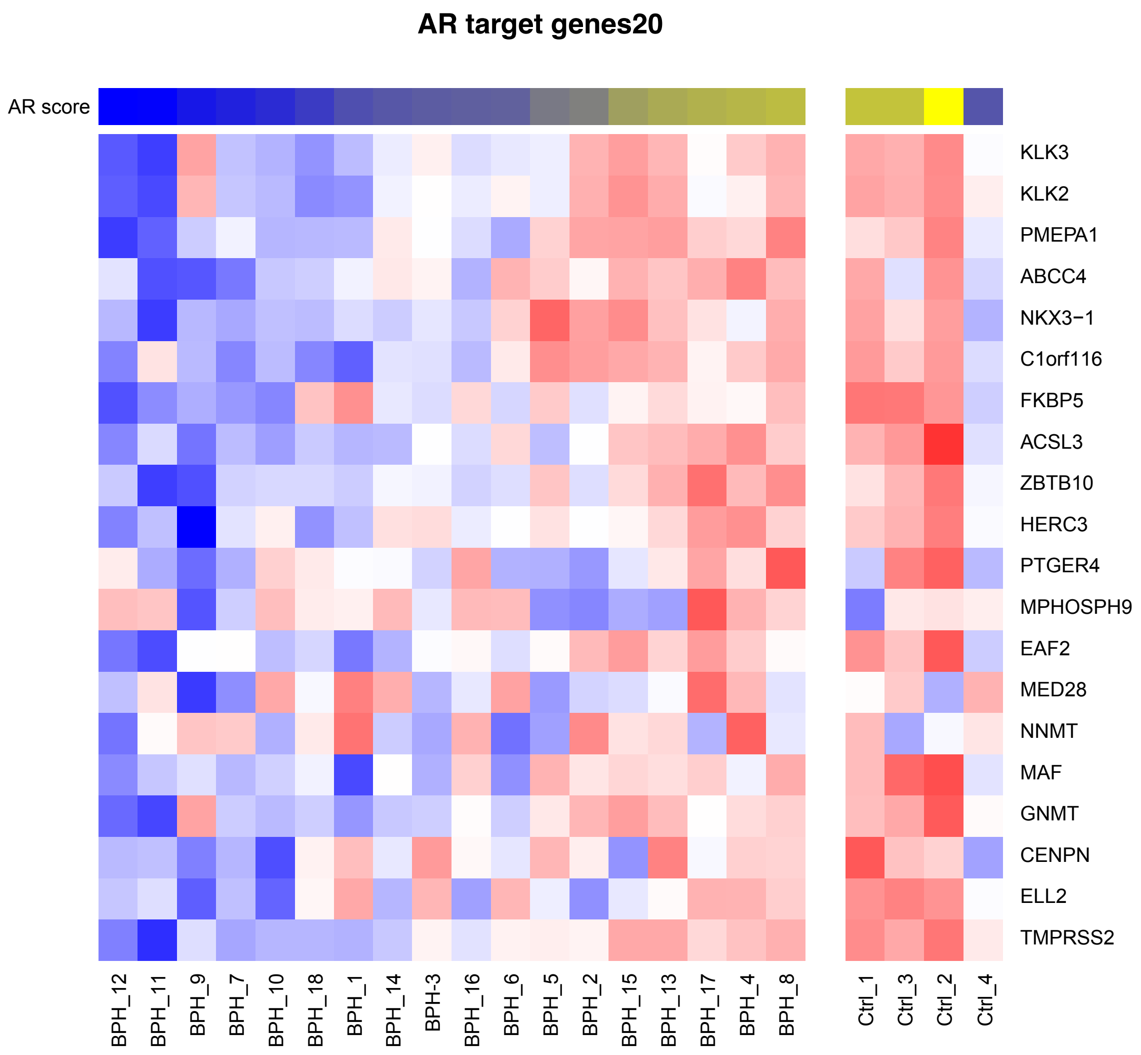

Supplementary Figure 7. Heatmap of BPH and control samples based on prostate AR target genes[2].

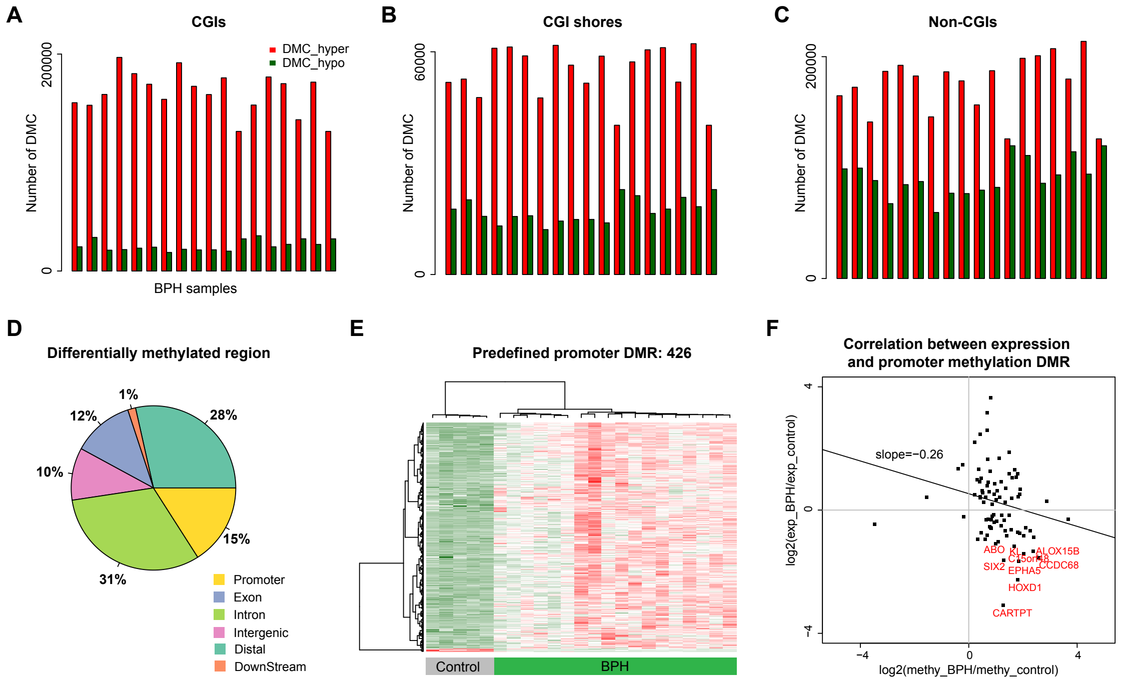

Supplementary Figure 8. Differentially methylation CpGs and regions found in BPH by ERRBS data.

(A) Hypermethylation domination found in CpG islands (CGIs). Red bar represents the hypermethylated CpGs, and green bar represents the hypomethylated CpGs.

(B) Hypermethylation domination found in CGI shores.

(C) Hypermethylation domination found in non-CGIs.

(D) Pie chart of differentially methylated regions between BPH and control samples among different genomic regions. Different colors denote different genomic related regions

(E) Hierarchical clustering and heatmap of DMRs in promoter between BPH and control samples.

(F) The negative correlation between transcription and methylation signatures, and the examples of epigenetically silent genes are shown in red color.

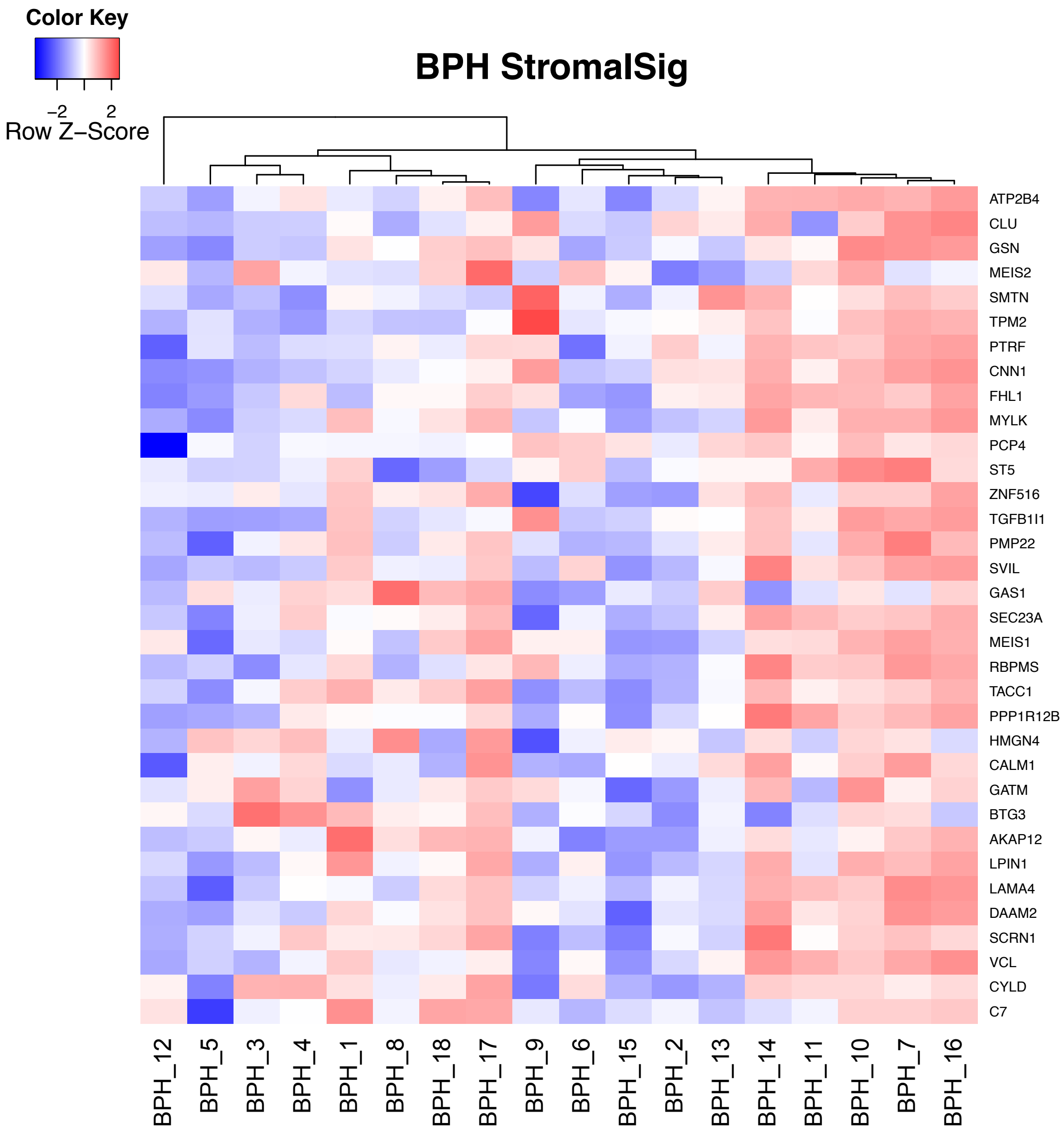

Supplementary Figure 9. Hierarchical clustering and heatmap of 18 BPH samples based on prostate stromal signature[3].

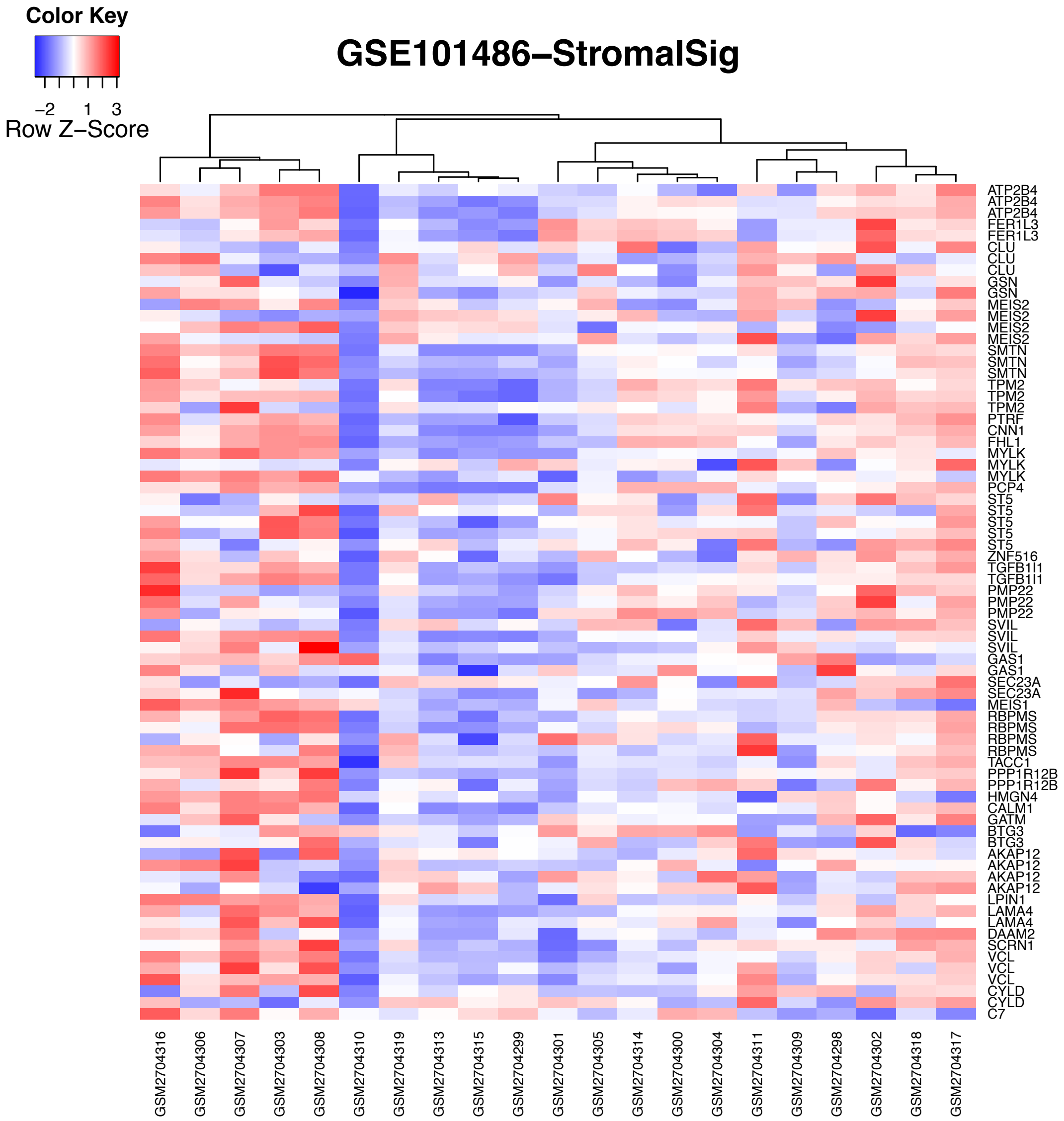

Supplementary Figure 10. Hierarchical clustering and heatmap of GSE101486 samples based on prostate stromal signature[3].

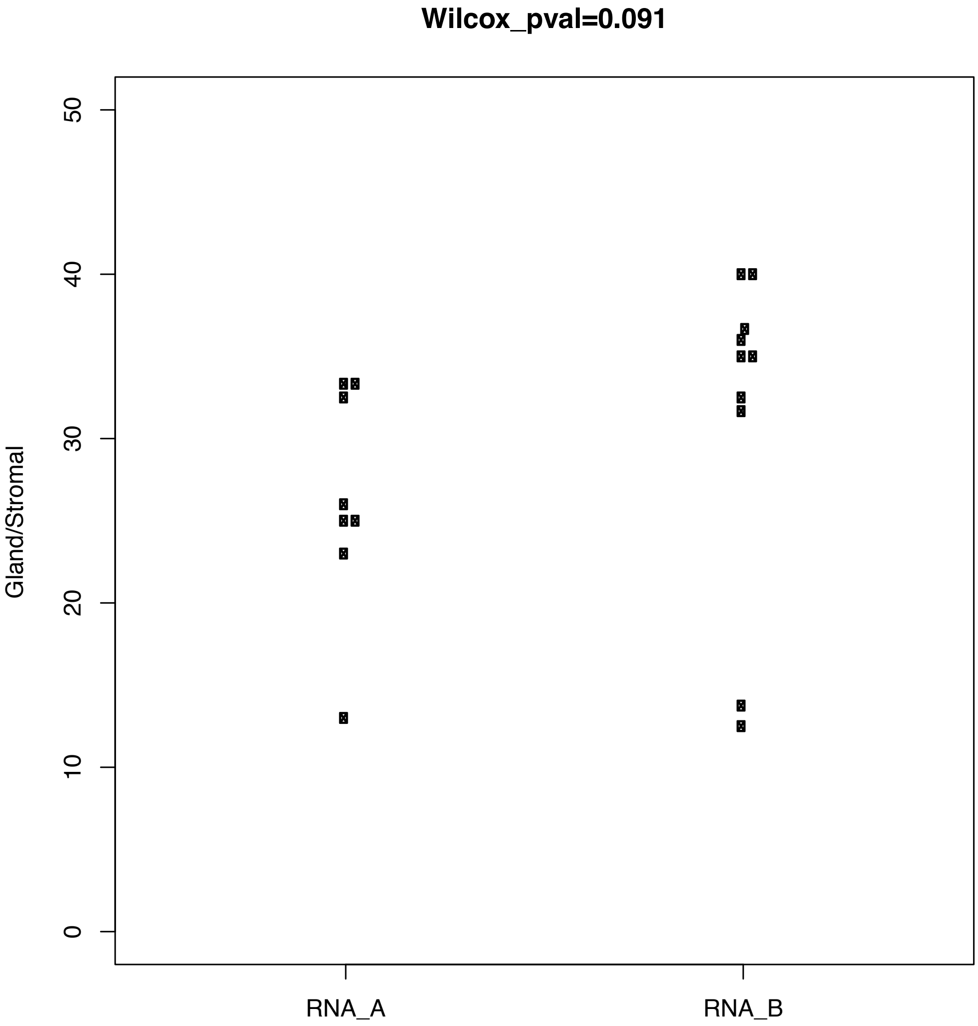

Supplementary Figure 11. Beeswarm plot of stromal enrichment difference between BPH subgroups. The y-axis denotes the average of grand/stromal cell ratio from multiple slides for each BPH sample. The lower ratio represents higher stromal enrichment. The comparison p-value was calculated from the grand/stromal cell ratio difference between two BPH subgroups via using two-sided Wilcoxon signed-rank test.

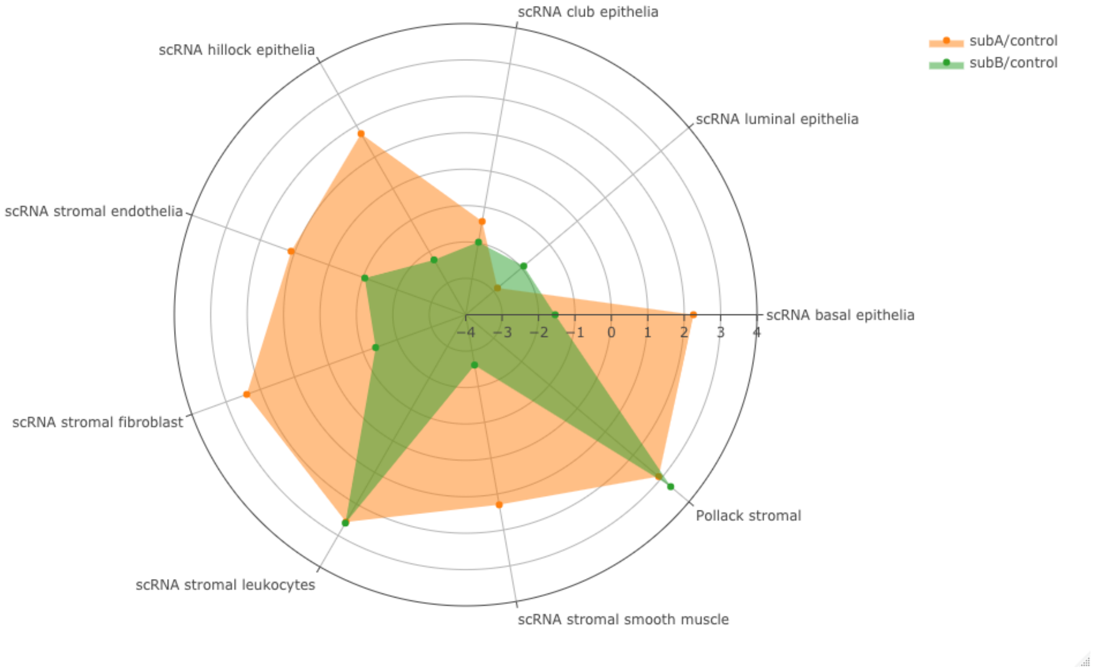

Supplementary Figure 12. Spider chart of normalized enrichment score of stromal signatures from single-cell RNA-seq[4] and bulk RNA-seq data[5] in BPH subgroups. Different colors represent different subgroups when compared to normal control samples.

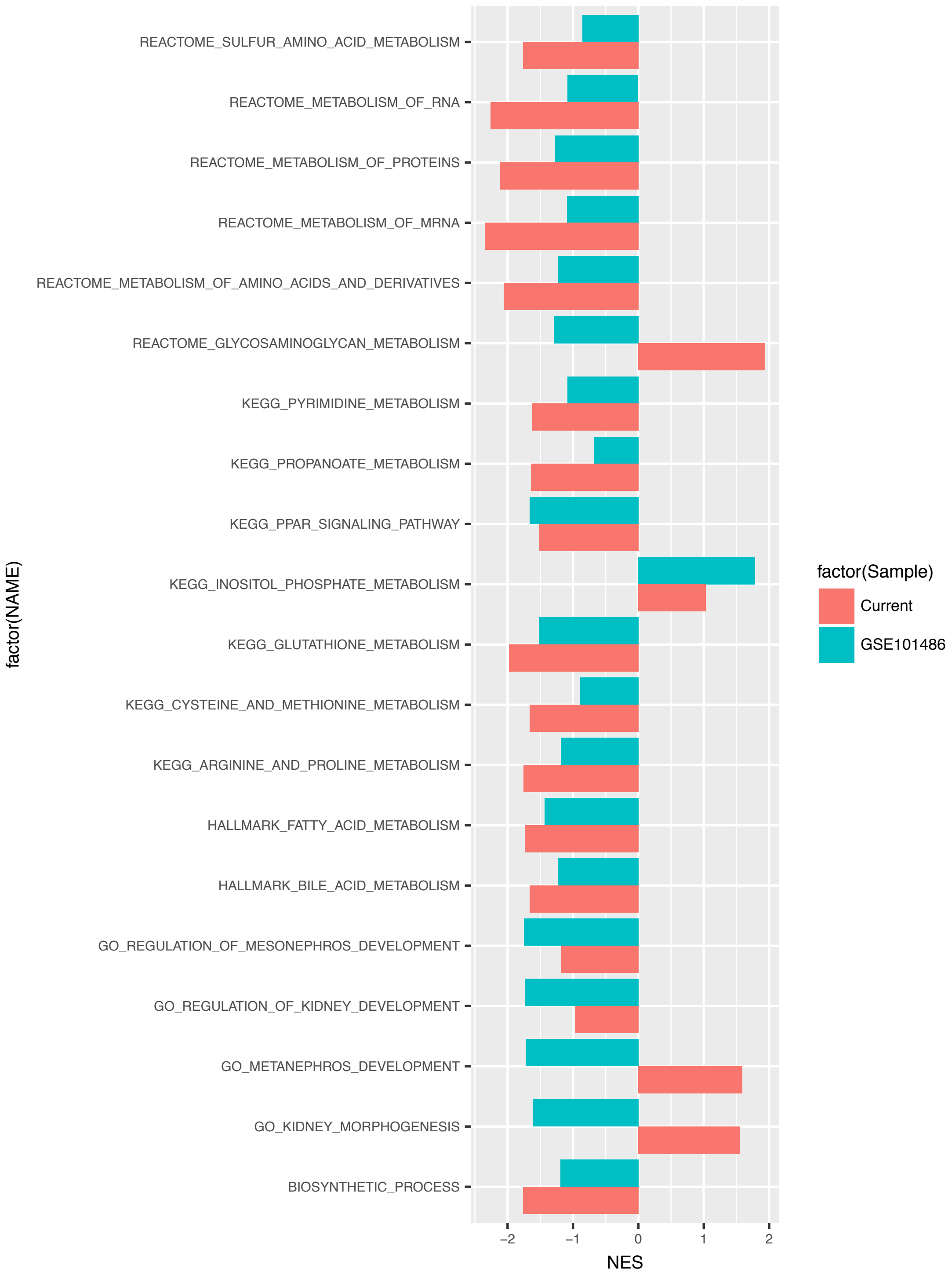

Supplementary Figure 13. Metabolism dysregulation between two subgroups from both current and GSE101486 studies via GSEA. The x-axis represents the normalized enrichment score, and different colors represent different studies.

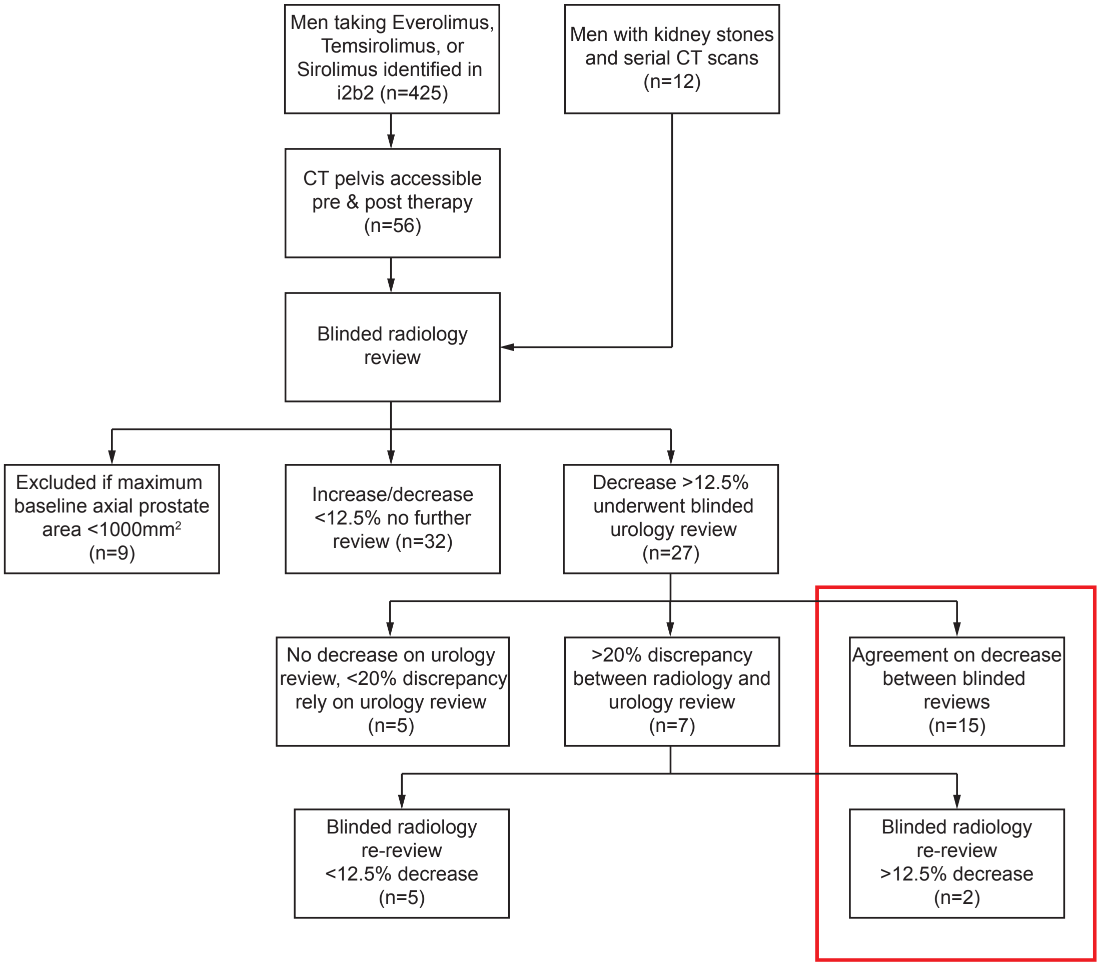

Supplementary Figure 14. Figure. CONSORT diagram detailing workflow for clinical study. Box denotes patients considered to have a significant decrease in prostate size.

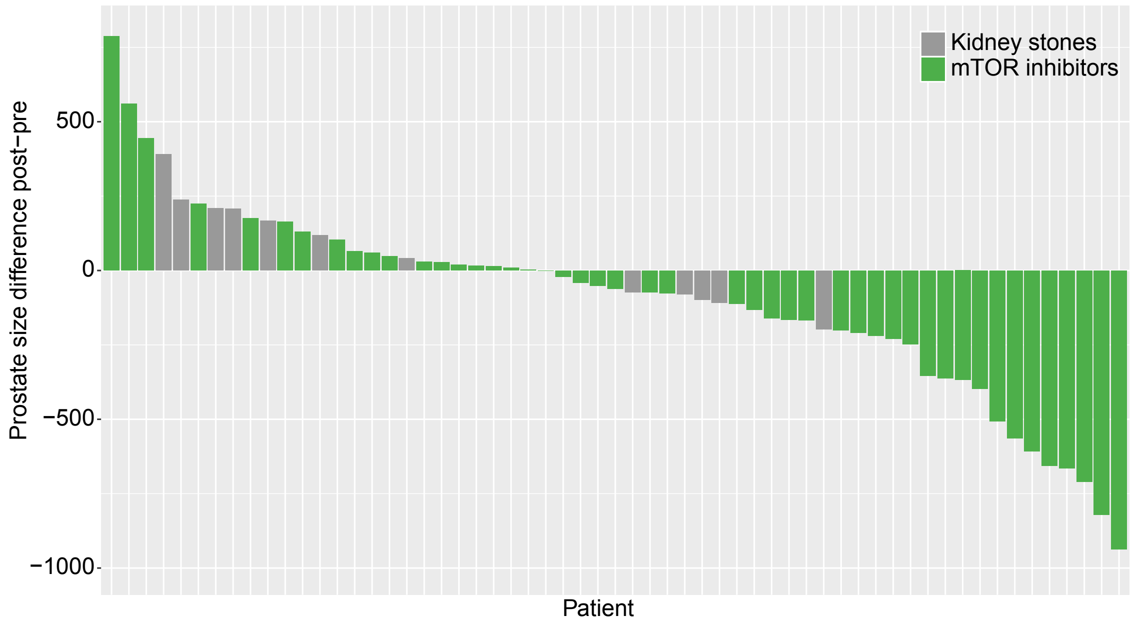

Supplementary Figure 15. Boxplot of prostate size changes in axial area on patients with and without *mTOR* inhibitors. Different color represents different treatment.

Reference

1. Cancer Genome Atlas Research N. The Molecular Taxonomy of Primary Prostate Cancer. *Cell* **163**, 1011-1025 (2015).

2. Hieronymus H*, et al.* Gene expression signature-based chemical genomic prediction identifies a novel class of HSP90 pathway modulators. *Cancer cell* **10**, 321-330 (2006).

3. Tomlins SA*, et al.* Integrative molecular concept modeling of prostate cancer progression. *Nature genetics* **39**, 41-51 (2007).

4. Henry GH*, et al.* A Cellular Anatomy of the Normal Adult Human Prostate and Prostatic Urethra. *Cell reports* **25**, 3530-3542 e3535 (2018).

5. Middleton LW*, et al.* Genomic analysis of benign prostatic hyperplasia implicates cellular re-landscaping in disease pathogenesis. *JCI insight* **5**, (2019).

6. Chakravarty D*, et al.* The oestrogen receptor alpha-regulated lncRNA NEAT1 is a critical modulator of prostate cancer. *Nature communications* **5**, 5383 (2014).

7. Lin PC*, et al.* Epigenomic alterations in localized and advanced prostate cancer. *Neoplasia* **15**, 373-383 (2013).

8. Beltran H*, et al.* Molecular characterization of neuroendocrine prostate cancer and identification of new drug targets. *Cancer discovery* **1**, 487-495 (2011).
