# Supplementary material for: Integrative multiplatform molecular profiling of benign prostatic hyperplasia identifies distinct subtypes
Source: Nat Commun. 2020 Apr 24;11:1987. doi: 10.1038/s41467-020-15913-6 (PMC7181734; doi:10.1038/s41467-020-15913-6)
Supplement: Supplementary file 3 — Description of Additional Supplementary Files [file 41467_2020_15913_MOESM3_ESM.docx]

**Description of Additional Supplementary Files**

**Supplementary Dataset 1 (Supplementary Table 1):** Clinical annotations of BPH samples.

**Supplementary Dataset 2 (Supplementary Table 2):** Mutation results of BPH samples.

**Supplementary Dataset 3 (Supplementary Table 3):** Small indel results of BPH samples.

**Supplementary Dataset 4 (Supplementary Table 4):** Recurrent amplifications of BPH samples.

**Supplementary Dataset 5 (Supplementary Table 5):** Recurrent deletions of BPH samples.

**Supplementary Dataset 6 (Supplementary Table 6):** No known mutations from primary prostate cancer found in BPH WGS/WES data.

**Supplementary Dataset 7 (Supplementary Table 7):** Clinical information of RNA-seq control samples.

**Supplementary Dataset 8 (Supplementary Table 8):** No known mutations from primary prostate cancer found in BPH RNA-seq data.

**Supplementary Dataset 9 (Supplementary Table 9):** Transcriptional signature between BPH and control samples.

**Supplementary Dataset 10 (Supplementary Table 10):** Promoter methylation signature between BPH and control samples.

**Supplementary Dataset 11 (Supplementary Table 11):** Transcriptional signature between two BPH subgroups.

**Supplementary Dataset 12 (Supplementary Table 12):** GSEA output of metabolism related signatures from subgroup BPH-A when compared to the other subgroup from current study, and from one subgroup when compared to the other subgroups from GSE101486 study.

**Supplementary Dataset 13 (Supplementary Table 13):** Transcriptional signature between BPH-A subgroup and control samples.

**Supplementary Dataset 14 (Supplementary Table 14):** Transcriptional signature between BPH-B subgroup and control samples.

**Supplementary Dataset 15 (Supplementary Table 15):** GSEA output difference of hallmark signatures from two BPH subgroups when compared to control samples.

**Supplementary Dataset 16 (Supplementary Table 16:** Nominated compounds from each BPH subgroup when comparing with control samples across multiple cell lines, and summary from all cell lines via Connectivity Map (CMAP).
