# Supplementary material for: Integrative multiplatform molecular profiling of benign prostatic hyperplasia identifies distinct subtypes
Source: Nat Commun. 2020 Apr 24;11:1987. doi: 10.1038/s41467-020-15913-6 (PMC7181734; doi:10.1038/s41467-020-15913-6)
Supplement: Supplementary file 20 — Reporting Summary [file 41467_2020_15913_MOESM20_ESM.pdf]

nature research

Corresponding author(s): Christopher E. Barbieri

Last updated by author(s): Jan 17, 2020

# Reporting Summary

Nature Research wishes to improve the reproducibility of the work that we publish. This form provides structure for consistency and transparency in reporting. For further information on Nature Research policies, see Authors & Referees and the Editorial Policy Checklist .

## Statistics

For all statistical analyses, confirm that the following items are present in the figure legend, table legend, main text, or Methods section.

| n/a | Confirmed | |
|---|---|---|
| ☐ | ☒ | The exact sample size ($n$) for each experimental group/condition, given as a discrete number and unit of measurement |
| ☐ | ☒ | A statement on whether measurements were taken from distinct samples or whether the same sample was measured repeatedly |
| ☐ | ☒ | The statistical test(s) used AND whether they are one- or two-sided<br>*Only common tests should be described solely by name; describe more complex techniques in the Methods section.* |
| ☒ | ☐ | A description of all covariates tested |
| ☒ | ☐ | A description of any assumptions or corrections, such as tests of normality and adjustment for multiple comparisons |
| ☐ | ☒ | A full description of the statistical parameters including central tendency (e.g. means) or other basic estimates (e.g. regression coefficient) AND variation (e.g. standard deviation) or associated estimates of uncertainty (e.g. confidence intervals) |
| ☐ | ☒ | For null hypothesis testing, the test statistic (e.g. $F$, $t$, $r$) with confidence intervals, effect sizes, degrees of freedom and $P$ value noted<br>*Give P values as exact values whenever suitable.* |
| ☒ | ☐ | For Bayesian analysis, information on the choice of priors and Markov chain Monte Carlo settings |
| ☒ | ☐ | For hierarchical and complex designs, identification of the appropriate level for tests and full reporting of outcomes |
| ☒ | ☐ | Estimates of effect sizes (e.g. Cohen's $d$, Pearson's $r$), indicating how they were calculated |

*Our web collection on statistics for biologists contains articles on many of the points above.*

## Software and code

Policy information about availability of computer code

**Data collection**

HiSeq 2500 was used for whole exome sequencing (WES), RNA-seq and Enhanced Reduced Representation Bisulfite Sequencing (ERRBS) in the Genomics and Epigenomics Core of Weill Cornell Medicine. HiSeq 4000 was used for whole genome sequencing (WGS) in New York Genome Center. DNA from blood tissue were analyzed by Affymetrix SNP 6.0 arrays to detect copy number alterations. The "gdc-client" tool was used to download mutations and copy number alterations results from the TCGA GDC data portal.

**Data analysis**

Paired-end sequence reads of WGS and WES data were aligned to the human reference genome (hg19) using BWA v0.7.12. Sorted bam files were generate via SAMtools v.0.1.19, and the duplicated mapped reads were marked with Picard v1.134. BAM files were locally realigned to the human reference genome using GATK v3.7, and somatic base substitutions and small indels were detected by using MuTect v1.1.4 and Varscan2 v2.3.9, with the sequencing coverage cutoff of at least 14x in BPH and 8x in control samples. Mutations were defined as the shared output between MuTect and Varscan2. After excluding the known human SNPs (dbSNP Build 150) and SNPs detected from control samples, the remaining mutations were annotated by ANNOVAR v2018.04.16 with GENCODE human gene annotation. The mutation signature was detected by using SomaticSignatures v2.20.0. DELLY v0.8.1, BreakDancer v1.3.6 and CREST v2016.12.07 were used to identify the genomic translocations.

Quality control, segmentation and copy number analysis were performed. Segments with log2-ratio >0.3 were defined as genomic amplifications, and log2-ratio <-0.3 were defined as genomic deletions.

RNA-seq reads were mapped to the human reference genome sequence (hg19) using STAR v2.4.0j. Then the resulting BAM files were subsequently converted into mapped-read format (MRF) using RSEQtools v0.6. The read count of each gene was calculated via HTSeq v0.11.1 using GENCODE as reference gene annotation set. Quantification of gene expression was performed via RSEQtools v0.6, and expression levels (RPKM) were estimated by counting all nucleotides mapped to each gene and were normalized by the total number of mapped nucleotides (per million) and the gene length (per kb). Fusion genes were detected via FusionSeq v0.1.2. Combat v3.20.0 was used to remove the batch effect of different RNA-seq libraries for the downstream gene expression analysis. Heatmap and hierarchical clustering were performed via using correlation distance and Ward's method. GSEA v3.0 was performed using JAVA program and run in pre-ranked mode to identify enriched signatures. The GSEA plot, normalized enrichment score and q-values were derived from GSEA

output for hallmark signature, and the metabolism related signatures were derived from MSigDB v6.2 database. Differential expression analysis was performed using the Wilcoxon signed-rank and F-statistic test after transforming the RPKMs via log2(RPKM + 1). Multiple-hypothesis testing was considered by using Benjamini-Hochberg (BH; FDR) correction. The immune score and stromal score were calculated from gene expression of BPH samples via ESTIMATE v2.0.0. The ImmuneSum was defined as the sum of normalized z-scores from gene expression of immune markers including PDCD1, PDCD1LG2, CD274, CD8A and CD8B. The comparison of molecular and clinical features between two subgroups was performed using Fisher's exact test and Wilcoxon signed-rank test. The compounds for each subgroup were identified by using Connectivity Map (CMAP) with the top most overexpressed and underexpressed genes as the input, and CMAP score >90 were used to select the nominated compounds.

For the ERRBS analysis, Differentially methylated CpGs (DMCs) were identified by methylKit and RRBSeeqer (false discovery rate =5%, and methylation difference more than 10%). Differentially methylated regions (DMRs) were defined as regions containing at least five DMCs within 250bp window. Genomic regions for CpGs were defined according to the following definitions. CGIs (CpG islands) were defined using annotations from RefSeq. CGI shores were defined as the regions encompassing 1kb upstream and downstream of known CGIs. Non-CGIs were defined as regions at least 10kb away from known CGIs. Promoters were defined as the regions encompassing 2kb upstream and downstream of the TSS (transcription start site) of RefSeq genes. Promoter methylation for each gene was calculated by averaging the methylation levels of all CpGs covered in the promoter.

For manuscripts utilizing custom algorithms or software that are central to the research but not yet described in published literature, software must be made available to editors/reviewers. We strongly encourage code deposition in a community repository (e.g. GitHub). See the Nature Research guidelines for submitting code & software for further information.

## Data

Policy information about availability of data

All manuscripts must include a data availability statement. This statement should provide the following information, where applicable:

- Accession codes, unique identifiers, or web links for publicly available datasets
- A list of figures that have associated raw data
- A description of any restrictions on data availability

The SNP array data has been deposited in GEO under the accession GSE124187, RNA-seq data has been deposited in GEO under the accession GSE132714, and ERRBS data has been deposited in GEO under the accession GSE123111.

# Field-specific reporting

Please select the one below that is the best fit for your research. If you are not sure, read the appropriate sections before making your selection.

☒ Life sciences ☐ Behavioural & social sciences ☐ Ecological, evolutionary & environmental sciences

For a reference copy of the document with all sections, see nature.com/documents/nr-reporting-summary-flat.pdf

# Life sciences study design

All studies must disclose on these points even when the disclosure is negative.

| | |
|---|---|
| Sample size | 18 patients with BPH (benign prostatic hyperplasia) were enrolled for sequencing of prostate tissue samples in this project. |
| Data exclusions | No exclusions |
| Replication | No replication |
| Randomization | No randomization. This is a retrospective cohort study. |
| Blinding | No blinding This is a retrospective cohort study. |

# Reporting for specific materials, systems and methods

We require information from authors about some types of materials, experimental systems and methods used in many studies. Here, indicate whether each material, system or method listed is relevant to your study. If you are not sure if a list item applies to your research, read the appropriate section before selecting a response.

## Materials & experimental systems

| n/a | Involved in the study |
| --- | --- |
| ☐ | ☐ Antibodies |
| ☐ | ☐ Eukaryotic cell lines |
| ☐ | ☐ Palaeontology |
| ☐ | ☐ Animals and other organisms |
| ☐ | ☒ Human research participants |
| ☐ | ☐ Clinical data |

## Methods

| n/a | Involved in the study |
| --- | --- |
| ☐ | ☐ ChIP-seq |
| ☐ | ☐ Flow cytometry |
| ☐ | ☐ MRI-based neuroimaging |

# Antibodies

| | |
| --- | --- |
| Antibodies used | *Describe all antibodies used in the study; as applicable, provide supplier name, catalog number, clone name, and lot number.* |
| Validation | *Describe the validation of each primary antibody for the species and application, noting any validation statements on the manufacturer's website, relevant citations, antibody profiles in online databases, or data provided in the manuscript.* |

# Eukaryotic cell lines

Policy information about cell lines

| | |
| --- | --- |
| Cell line source(s) | *State the source of each cell line used.* |
| Authentication | *Describe the authentication procedures for each cell line used OR declare that none of the cell lines used were authenticated.* |
| Mycoplasma contamination | *Confirm that all cell lines tested negative for mycoplasma contamination OR describe the results of the testing for mycoplasma contamination OR declare that the cell lines were not tested for mycoplasma contamination.* |
| Commonly misidentified lines (See ICLAC register) | *Name any commonly misidentified cell lines used in the study and provide a rationale for their use.* |

# Palaeontology

| | |
| --- | --- |
| Specimen provenance | *Provide provenance information for specimens and describe permits that were obtained for the work (including the name of the issuing authority, the date of issue, and any identifying information).* |
| Specimen deposition | *Indicate where the specimens have been deposited to permit free access by other researchers.* |
| Dating methods | *If new dates are provided, describe how they were obtained (e.g. collection, storage, sample pretreatment and measurement), where they were obtained (i.e. lab name), the calibration program and the protocol for quality assurance OR state that no new dates are provided.* |

☐ Tick this box to confirm that the raw and calibrated dates are available in the paper or in Supplementary Information.

# Animals and other organisms

Policy information about studies involving animals; ARRIVE guidelines recommended for reporting animal research

| | |
| --- | --- |
| Laboratory animals | *For laboratory animals, report species, strain, sex and age OR state that the study did not involve laboratory animals.* |
| Wild animals | *Provide details on animals observed in or captured in the field; report species, sex and age where possible. Describe how animals were caught and transported and what happened to captive animals after the study (if killed, explain why and describe method; if released, say where and when) OR state that the study did not involve wild animals.* |
| Field-collected samples | *For laboratory work with field-collected samples, describe all relevant parameters such as housing, maintenance, temperature, photoperiod and end-of-experiment protocol OR state that the study did not involve samples collected from the field.* |
| Ethics oversight | *Identify the organization(s) that approved or provided guidance on the study protocol, OR state that no ethical approval or guidance was required and explain why not.* |

Note that full information on the approval of the study protocol must also be provided in the manuscript.

# Human research participants

Policy information about studies involving human research participants

| Field | Response |
|---|---|
| Population characteristics | Patients with BPH (benign prostatic hyperplasia) were prospectively enrolled for sequencing of prostate tissue samples from transitional zones . The mean age for BPH cases was 69, and all BPH patients were previously been treated with medical therapy before surgery. |
| Recruitment | We selected samples from patients with very large prostates (greater than 100cc and top 1 percentile ), based on the rationale that these extreme outliers were more likely to harbor biologically informative events. |
| Ethics oversight | This study was approved by the institutional review board of Weill Cornell Medical College. Written informed consent was obtained, including discussion of risks associated with germline sequencing. |

Note that full information on the approval of the study protocol must also be provided in the manuscript.

# Clinical data

Policy information about clinical studies

All manuscripts should comply with the ICMJEguidelines for publication of clinical research and a completedCONSORT checklist must be included with all submissions.

| Field | Response |
|---|---|
| Clinical trial registration | *Provide the trial registration number from ClinicalTrials.gov or an equivalent agency.* |
| Study protocol | *Note where the full trial protocol can be accessed OR if not available, explain why.* |
| Data collection | *Describe the settings and locales of data collection, noting the time periods of recruitment and data collection.* |
| Outcomes | *Describe how you pre-defined primary and secondary outcome measures and how you assessed these measures.* |

# ChIP-seq

## Data deposition

☐ Confirm that both raw and final processed data have been deposited in a public database such as GEO.

☐ Confirm that you have deposited or provided access to graph files (e.g. BED files) for the called peaks.

| Field | Response |
|---|---|
| Data access links<br>*May remain private before publication.* | *For "Initial submission" or "Revised version" documents, provide reviewer access links. For your "Final submission" document, provide a link to the deposited data.* |
| Files in database submission | *Provide a list of all files available in the database submission.* |
| Genome browser session<br>(e.g. UCSC) | *Provide a link to an anonymized genome browser session for "Initial submission" and "Revised version" documents only, to enable peer review. Write "no longer applicable" for "Final submission" documents.* |

## Methodology

| Field | Response |
|---|---|
| Replicates | *Describe the experimental replicates, specifying number, type and replicate agreement.* |
| Sequencing depth | *Describe the sequencing depth for each experiment, providing the total number of reads, uniquely mapped reads, length of reads and whether they were paired- or single-end.* |
| Antibodies | *Describe the antibodies used for the ChIP-seq experiments; as applicable, provide supplier name, catalog number, clone name, and lot number.* |
| Peak calling parameters | *Specify the command line program and parameters used for read mapping and peak calling, including the ChIP, control and index files used.* |
| Data quality | *Describe the methods used to ensure data quality in full detail, including how many peaks are at FDR 5% and above 5-fold enrichment.* |
| Software | *Describe the software used to collect and analyze the ChIP-seq data. For custom code that has been deposited into a community repository, provide accession details.* |

## Flow Cytometry

### Plots

Confirm that:

☐ The axis labels state the marker and fluorochrome used (e.g. CD4-FITC).

☐ The axis scales are clearly visible. Include numbers along axes only for bottom left plot of group (a 'group' is an analysis of identical markers).

☐ All plots are contour plots with outliers or pseudocolor plots.

☐ A numerical value for number of cells or percentage (with statistics) is provided.

### Methodology

| | |
|---|---|
| Sample preparation | *Describe the sample preparation, detailing the biological source of the cells and any tissue processing steps used.* |
| Instrument | *Identify the instrument used for data collection, specifying make and model number.* |
| Software | *Describe the software used to collect and analyze the flow cytometry data. For custom code that has been deposited into a community repository, provide accession details.* |
| Cell population abundance | *Describe the abundance of the relevant cell populations within post-sort fractions, providing details on the purity of the samples and how it was determined.* |
| Gating strategy | *Describe the gating strategy used for all relevant experiments, specifying the preliminary FSC/SSC gates of the starting cell population, indicating where boundaries between "positive" and "negative" staining cell populations are defined.* |

☐ Tick this box to confirm that a figure exemplifying the gating strategy is provided in the Supplementary Information.

## Magnetic resonance imaging

### Experimental design

| | |
|---|---|
| Design type | *Indicate task or resting state; event-related or block design.* |
| Design specifications | *Specify the number of blocks, trials or experimental units per session and/or subject, and specify the length of each trial or block (if trials are blocked) and interval between trials.* |
| Behavioral performance measures | *State number and/or type of variables recorded (e.g. correct button press, response time) and what statistics were used to establish that the subjects were performing the task as expected (e.g. mean, range, and/or standard deviation across subjects).* |

### Acquisition

| | |
|---|---|
| Imaging type(s) | *Specify: functional, structural, diffusion, perfusion.* |
| Field strength | *Specify in Tesla* |
| Sequence & imaging parameters | *Specify the pulse sequence type (gradient echo, spin echo, etc.), imaging type (EPI, spiral, etc.), field of view, matrix size, slice thickness, orientation and TE/TR/flip angle.* |
| Area of acquisition | *State whether a whole brain scan was used OR define the area of acquisition, describing how the region was determined.* |

Diffusion MRI ☐ Used ☐ Not used

### Preprocessing

| | |
|---|---|
| Preprocessing software | *Provide detail on software version and revision number and on specific parameters (model/functions, brain extraction, segmentation, smoothing kernel size, etc.).* |
| Normalization | *If data were normalized/standardized, describe the approach(es): specify linear or non-linear and define image types used for transformation OR indicate that data were not normalized and explain rationale for lack of normalization.* |
| Normalization template | *Describe the template used for normalization/transformation, specifying subject space or group standardized space (e.g. original Talairach, MNI305, ICBM152) OR indicate that the data were not normalized.* |
| Noise and artifact removal | *Describe your procedure(s) for artifact and structured noise removal, specifying motion parameters, tissue signals and physiological signals (heart rate, respiration).* |
| Volume censoring | *Define your software and/or method and criteria for volume censoring, and state the extent of such censoring.* |

## Statistical modeling & inference

| | |
|---|---|
| Model type and settings | *Specify type (mass univariate, multivariate, RSA, predictive, etc.) and describe essential details of the model at the first and second levels (e.g. fixed, random or mixed effects; drift or auto-correlation).* |
| Effect(s) tested | *Define precise effect in terms of the task or stimulus conditions instead of psychological concepts and indicate whether ANOVA or factorial designs were used.* |

Specify type of analysis: ☐ Whole brain ☐ ROI-based ☐ Both

| | |
|---|---|
| Statistic type for inference<br>(See Eklund et al. 2016) | *Specify voxel-wise or cluster-wise and report all relevant parameters for cluster-wise methods.* |
| Correction | *Describe the type of correction and how it is obtained for multiple comparisons (e.g. FWE, FDR, permutation or Monte Carlo).* |

## Models & analysis

| n/a | Involved in the study |
|---|---|
| ☐ | ☐ Functional and/or effective connectivity |
| ☐ | ☐ Graph analysis |
| ☐ | ☐ Multivariate modeling or predictive analysis |

| | |
|---|---|
| Functional and/or effective connectivity | *Report the measures of dependence used and the model details (e.g. Pearson correlation, partial correlation, mutual information).* |
| Graph analysis | *Report the dependent variable and connectivity measure, specifying weighted graph or binarized graph, subject- or group-level, and the global and/or node summaries used (e.g. clustering coefficient, efficiency, etc.).* |
| Multivariate modeling and predictive analysis | *Specify independent variables, features extraction and dimension reduction, model, training and evaluation metrics.* |
